# Modification of G-C_3_N_4_ by the Surface Alkalinization Method and Its Photocatalytic Depolymerization of Lignin

**DOI:** 10.3390/ma18143350

**Published:** 2025-07-17

**Authors:** Zhongmin Ma, Ling Zhang, Lihua Zang, Fei Yu

**Affiliations:** School of Environmental Science and Engineering, Qilu University of Technology (Shandong Academy of Sciences), Jinan 250353, China; amzm365@163.com (Z.M.); zl17354612763@163.com (L.Z.)

**Keywords:** g-C_3_N_4_, photocatalytic, lignin, alkalinization, depolymerization

## Abstract

The efficient depolymerization of lignin has become a key challenge in the preparation of high-value-added chemicals. Graphitic carbon nitride (g-C_3_N_4_)-based photocatalytic system shows potential due to its mild and green characteristics over other depolymerization methods. However, its inherent defects, such as a wide band gap and rapid carrier recombination, severely limit its catalytic performance. In this paper, a g-C_3_N_4_ modification strategy of K⁺ doping and surface alkalinization is proposed, which is firstly applied to the photocatalytic depolymerization of the lignin β-O-4 model compound (2-phenoxy-1-phenylethanol). K⁺ doping is achieved by introducing KCl in the precursor thermal polymerization stage to weaken the edge structure strength of g-C_3_N_4_, and post-treatment with KOH solution is combined to optimize the surface basic groups. The structural/compositional evolution of the materials was analyzed by XRD, FTIR, and XPS. The morphology/element distribution was visualized by SEM-EDS, and the optoelectronic properties were evaluated by UV–vis DRS, PL, EIS, and transient photocurrent (TPC). K⁺ doping and surface alkalinization synergistically regulate the layered structure of the material, significantly increase the specific surface area, introduce nitrogen vacancies and hydroxyl functional groups, effectively narrow the band gap (optimized to 2.35 eV), and inhibit the recombination of photogenerated carriers by forming electron capture centers. Photocatalytic experiments show that the alkalinized g-C_3_N_4_ can completely depolymerize 2-phenoxy-1-phenylethanol with tunable product selectivity. By adjusting reaction time and catalyst dosage, the dominant product can be shifted from benzaldehyde (up to 77.28% selectivity) to benzoic acid, demonstrating precise control over oxidation degree. Mechanistic analysis shows that the surface alkaline sites synergistically optimize the C_β_-O bond breakage path by enhancing substrate adsorption and promoting the generation of active oxygen species (·OH, ·O_2_^−^). This study provides a new idea for the efficient photocatalytic depolymerization of lignin and lays an experimental foundation for the interface engineering and band regulation strategies of g-C_3_N_4_-based catalysts.

## 1. Introduction

Against the backdrop of the increasingly severe global environmental and energy crisis, lignin, as one of the most abundant renewable aromatic polymers on Earth, has attracted widespread attention due to its potential to replace petrochemical resources and its conversion into high-value chemicals [1,2]. However, the complex and highly cross-linked three-dimensional structure of lignin necessitates harsh reaction conditions (e.g., high temperatures and pressures) in conventional degradation processes, resulting thus in high energy consumption, poor selectivity, and low yields of target products [3,4]. Consequently, developing mild, efficient, and environmentally friendly degradation technologies has emerged as a critical research focus.

Photocatalytic technology is regarded as a promising solution for efficient lignin depolymerization, as it enables reactions under ambient temperature and pressure while offering significant advantages, including environmental benignity and low energy consumption [5]. Recent years have witnessed growing interest in photocatalytic technology for biomass valorization, particularly lignin conversion, owing to its advantages of mild reaction conditions, environmental benignity, and high product selectivity. In photocatalytic lignin depolymerization, photogenerated charge carriers and reactive species enable precise cleavage of targeted bonds while preserving functional groups, thereby yielding high-value aromatic monomers [6]. Although extensive research has focused on photocatalytic cleavage of C–O bonds in lignin, only a limited number of photocatalysts have demonstrated efficacy for selective C–C bond scission. While homogeneous systems employing vanadium-, iridium-, and cerium-based photocatalysts have been developed [7,8,9], technical challenges such as catalyst separation and recovery persist [10]. Among various photocatalysts, graphitic carbon nitride (g-C_3_N_4_), a metal-free organic semiconductor, exhibits notable application potential due to its abundant raw materials, facile synthesis, and excellent visible-light responsiveness. Wang et al. reported g-C_3_N_4_ as a photocatalyst capable of efficiently cleaving β-O-4 linkages into valuable aromatic products with up to 91% selectivity for C–C bond cleavage [11]. However, its insufficient photooxidation capability—attributed to a high valence band position and rapid charge carrier recombination—limits broader applicability [12]. Consequently, there is an urgent need to develop advanced photocatalysts that effectively accelerate C–C bond cleavage in β-O-4 lignin models with both high conversion efficiency and selectivity.

To address these limitations, surface engineering emerges as a pivotal strategy for enhancing photocatalytic performance by precisely tuning interfacial architectures and band structure alignment [13,14]. This approach concurrently establishes additional catalytic active sites while optimizing the separation kinetics and directional migration of photogenerated charge carriers, thereby significantly elevating the overall photocatalytic activity [15]. Specifically, constructing heterojunctions based on g-C_3_N_4_ facilitates spatial segregation of photogenerated electrons and holes across semiconductor interfaces through built-in electric fields [16], which effectively suppresses charge recombination by diverting carriers along Z-scheme or type-II pathways [17,18]. For instance, in g-C_3_N_4_-based heterojunctions (e.g., metal/g-C_3_N_4_, semiconductor/g-C_3_N_4_), the band offset drives electron transfer from higher to lower conduction bands while holes migrate inversely [16]. Moreover, incorporating a secondary phase significantly enhances photon capture efficiency, optimizes band alignment, and elevates surface reaction kinetics of g-C_3_N_4_. Based on distinct charge transfer mechanisms at the g-C_3_N_4_/cocatalyst interface, predominant heterojunction architectures encompass metal/g-C_3_N_4_, semiconductor/g-C_3_N_4_, carbonaceous/g-C_3_N_4_, and conductive polymer/g-C_3_N_4_ systems, establishing tailored charge transfer pathways for specific catalytic applications [19]. Among surface engineering strategies, alkaline functionalization has garnered significant attention owing to its operational simplicity and efficacy in photocatalytic enhancement [20,21,22]. Some studies demonstrate that alkaline treatment introduces hydroxyl groups, regulates electronic configuration, and refines band structure, thereby suppressing charge recombination and augmenting redox capabilities [23,24]. For instance, Gao et al. synthesized hydroxylated g-C_3_N_4_ via molten salt calcination of dicyandiamide/KCl/(NH_4_)_4_CO_3_ at 550 °C, which enhanced hole-trapping capability and significantly improved methyl orange degradation efficiency [25]. Separately, Wang et al. applied in situ hydroxylation using 0.001 M NaOH solution, achieving 2.3-fold higher photocatalytic hydrogen evolution compared to pristine g-C_3_N_4_ [26]. Furthermore, Xu et al. integrated Fe-doping with alkaline surface functionalization, enabling efficient visible-light-driven tetracycline antibiotic degradation through synergistic carrier separation and OH radical generation [27].

Although alkaline surface functionalization has demonstrated proven efficacy in enhancing the photocatalytic activity of g-C_3_N_4_ through improved charge separation kinetics, optimized band alignment, and elevated surface nucleophilicity [22,28,29], its application in photocatalytic lignin depolymerization—particularly for selective cleavage of recalcitrant β-O-4 linkages under mild conditions—remains unexplored in extant literature [30]. This research gap persists despite the established capability of alkalinized g-C_3_N_4_ to facilitate critical bond-forming reactions and degrade persistent organic pollutants, suggesting untapped potential for lignin valorization via surface-engineered photocatalysis [21].

In this work, the alkaline surface functionalization to engineer g-C_3_N_4_ for photocatalytic depolymerization of lignin model compounds was systematically researched. Specifically, K⁺ ion doping was achieved by incorporating variable KCl ratios during precursor thermal polymerization, which modulated the structural stability of g-C_3_N_4_ edge sites through coordination-induced lattice distortion. Subsequent surface treatment with graded KOH concentrations enabled precise alkalinity regulation, augmenting surface nucleophilicity and hydroxyl group density.

To systematically elucidate the synergistic effects of KCl doping and KOH treatment on morphological evolution, electronic structure, and charge carrier dynamics, comprehensive characterization methods—including XRD, XPS, PL and EIS analysis—were employed. Finally, using 2-phenoxy-1-phenylethanol (a model compound representing the β-O-4 bond structure in lignin) as the substrate, the depolymerization performance of the alkaline g-C_3_N_4_ under visible light irradiation was investigated, and the catalytic mechanism was preliminarily discussed. This work not only introduces a new surface-engineered g-C_3_N_4_ photocatalyst for selective β-O-4 cleavage but also offers a noble-metal-free, green, and tunable strategy for lignin valorization, which could be extended to real lignin depolymerization and biomass upgrading processes.

## 2. Materials and Method

### 2.1. Chemicals

Melamine, ethanol, potassium chloride, sodium sulfate, acetonitrile, 2-phenoxy-1-phenylethanol, benzoic acid, benzaldehyde, benzyl formate, phenol, acetophenone, 2-phenoxy-1-acetophenone were purchased from Sinopharm Chemical Reagent Co., Ltd. (Shanghai, China). All the experimental water was purchased from Hangzhou Wahaha Group Co., Ltd. (Hangzhou, China). The potassium hydroxide was purchased from Macklin Reagent (Shanghai, China), and the Nafion solution was purchased from DuPont China (Shenzhen, China).

### 2.2. Materials Preparation

**Pristine g-C_3_N_4_ (CN-0/KCN-0):** Melamine (3.0 g) was thermally polymerized in an alumina crucible under static air (ramp: 5 °C·min^−1^ to 550 °C; isothermal hold: 4 h). The naturally cooled yellow agglomerate was manually ground into homogeneous powder using an agate mortar.

**Alkali-Functionalized g-C_3_N_4_ via Dual-Modification Strategy:** A mixture of KCl and melamine was dispersed in a binary solvent system (30 mL deionized water and 30 mL ethanol). After magnetically stirring for 12 h, the suspension was dried at 60 °C for 24 h in a forced-air oven. The resultant precipitate was homogenized via ball-milling to ensure uniform KCl-melamine integration. The composite was transferred to an alumina crucible and subjected to thermal polymerization in a muffle furnace under static air: ramped to 550 °C at 3 °C·min^−1^ (isothermal hold: 4 h). The cooled product was manually ground using an agate mortar. Subsequently, 3.0 g of the powder was immersed in 30 mL KOH solution with magnetic stirring for 12 h. The suspension underwent centrifugation followed by repeated washing with deionized water until neutral pH. The solid was dried at 60 °C for 12 h to yield alkali-functionalized kg-C_3_N_4_.

The synthesized materials were systematically designated according to the KCl: melamine molar ratio and KOH concentration, as defined in Table 1. The KOH concentration used was adapted from the method described by Yu et al [31], while the ratios of KCl to melamine were optimized based on our preliminary experiments to evaluate the effects of K^+^ doping and surface alkalinization on photocatalytic performance.

### 2.3. Characterization

The surface morphology and the localized elemental composition of the prepared particles were obtained using a Czech TESCAN MIRA LMS SEM (Brno, Czech Republic) equipped with EDX (Xplore). The particle crystallinity was characterized by an X’Pert PRO XRD (PANalytical, Almelo, The Netherlands) with a scan rate of 5° min^−1^. XPS analyses of given samples were conducted by a Kratos AXIS Ultra DLD XPS (Kratos Analytical, Manchester, UK), and the original binding energies were corrected according to the C1s peak at 284.8 eV. A curve-fitting program (XPS-peak 4.1) was used to fit the XPS results. Chemical functional groups of the materials were identified using a Nicolet iS5 spectrometer (Thermo Fisher Scientific, Waltham, MA, USA). Samples were prepared via the KBr pellet method and scanned over 4000 to 500 cm^−1^ at 4 cm^−1^ resolution, with wavenumber accuracy maintained at ±0.01 cm^−1^. Optical absorption properties were evaluated on a Shimadzu UV-3600 spectrophotometer (Shimadzu Corporation, Kyoto, Japan) with BaSO_4_ as a reflectance standard. Data were converted to absorption spectra via the Kubelka-Munk function. Charge carrier recombination behavior was analyzed using an Edinburgh Instruments FLS1000 spectrofluorometer (Livingston, UK) under 325 nm excitation, with slit widths set to 2 nm.

### 2.4. Photocatalytic Degradation of Lignin Model Compound

**Figure 1:**
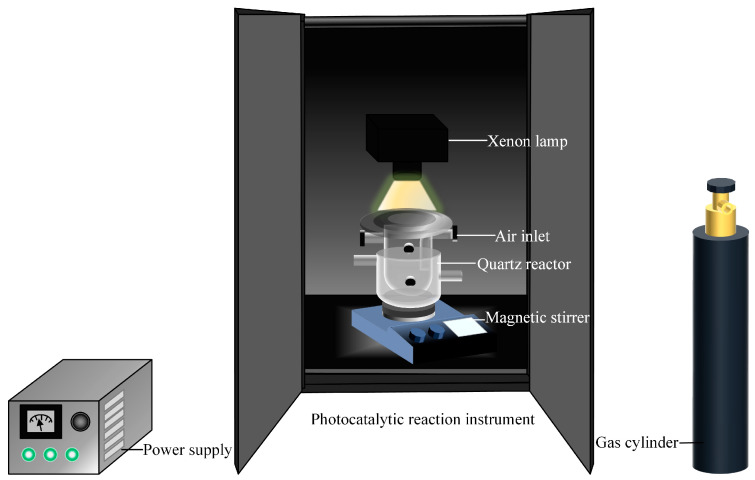
Schematic diagram of the photocatalytic reaction setup. A quartz reaction vessel equipped with a gas inlet and outlet was placed on a magnetic stirrer and irradiated with a Zhongjiao Jinyuan xenon lamp through a 420 nm cutoff filter. The setup was enclosed in a sealed photocatalytic reaction box under ambient atmosphere unless otherwise noted.

The photocatalytic system was established by combining 30 mg catalyst, 2 mg 2-phenoxy-1-phenylethanol, and 30 mL acetonitrile solvent in the reactor. The mixture was sonicated for 10 min under dark conditions, followed by magnetic stirring (15 min) to achieve homogeneous dispersion. Visible-light irradiation utilized a 300 W xenon lamp with a 420 nm cutoff filter to exclude ultraviolet wavelengths. A schematic illustration of the photocatalytic experimental setup is shown in Figure 1. During photocatalytic testing, 1 mL aliquots were extracted hourly and filtered through 0.22 μm membranes to remove catalyst. Quantitative analysis employed HPLC (Diamonsil C18 column: 4.6 × 250 mm, 5 μm; Guangzhou Green Baicao, Guangzhou, China) with UV detection at 230 nm, mobile phase acetonitrile/water (50%:50%, *v*/*v*) at 0.8 mL·min^−1^ flow rate, column temperature 30 °C, and injection volume 10 μL, enabling precise determination of model compound conversion efficiency and aromatic product generation rates.

The conversion rate of 2-phenoxy-1-phenylethanol and the molar yield of its product were quantified via Formulas (1) and (2).

(1) $$\mathrm{Conversion}\;\mathrm{Rate}=\frac{n_{m}\;-\;n_{r}}{n_{m}}\;\times \;100\%.$$

(2) $$\mathrm{Yield}=\frac{n_{p}}{n_{m}}\;\times \;100\%$$

The molar amounts are defined as *n*_r_ for the post-reaction lignin model compound, *n*_p_ for product p, and *n*_m_ for the initial lignin model compound. These parameters collectively quantify the reaction efficiency and product yield.

## 3. Results and Discussion

### 3.1. Characterization Results

**Figure 2:**
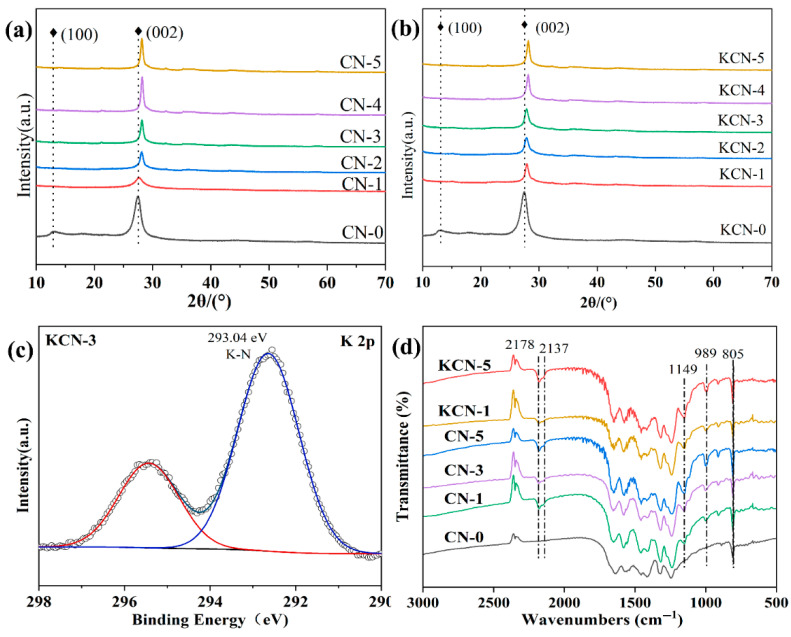
XRD patterns of (**a**) CN-X and (**b**) KCN-X, (**c**) XPS spectrum of KCN-3, and (**d**) FTIR spectra of different samples.

Figure 2a,b presented the XRD patterns of CN-X and KCN-X samples alongside pristine g-C_3_N_4_. The characteristic (100) and (002) planes of g-C_3_N_4_ were indexed at 13.0° and 27.5°, respectively. Notably, the (100) diffraction peak vanished in CN-X and KCN-X, while the (002) peak shifted toward higher angles, indicating reduced interlayer spacing (d-spacing). This structural perturbation correlates with the XPS signal at 293.04 eV (K 2p_3/2_) in KCN-3 (Figure 2c), consistent with K-N coordination. These observations collectively suggested K⁺ intercalation disrupts the in-plane periodicity of g-C_3_N_4_, thereby eliminating the (100) diffraction signature. Concerning dopant concentration effects, Figure 2a demonstrated that increasing KCl proportions progressively shifted the (002) peak to higher angles. CN-1 exhibited significantly diminished (002) peak intensity relative to g-C_3_N_4_, implying crystal lattice distortion. However, higher KCl ratios gradually enhance this peak’s intensity and sharpness, suggesting restored crystallinity. In contrast, Figure 2b revealed KOH concentration variations below 2 mol/L minimally alter the (002) peak position or intensity. Concentrations at 2 mol/L and 10 mol/L induce slight intensity increases, indicating structural changes predominantly occur during sintering rather than through subsequent alkalinization. FTIR spectra (Figure 2d) show that CN-X and KCN-X retained characteristic g-C_3_N_4_ vibrational modes post-alkalinization. However, emergent peaks at 1149 cm^−1^, 2137 cm^−1^, and 2178 cm^−1^ signified modified bonding environments. Specifically, peaks at 1149 cm^−1^ and 2137 cm^−1^ indicated potential replacement of terminal –NH groups by oxygen functionalities (e.g., C-OH/C=O), while the 2178 cm^−1^ peak suggested C≡N formation via C-N bond cleavage. The absence of N–H bands and the redshift of C=N/C-N vibrations collectively confirmed structural rearrangement during K-modification, ref. [22] corroborating XRD findings.

**Figure 3:**
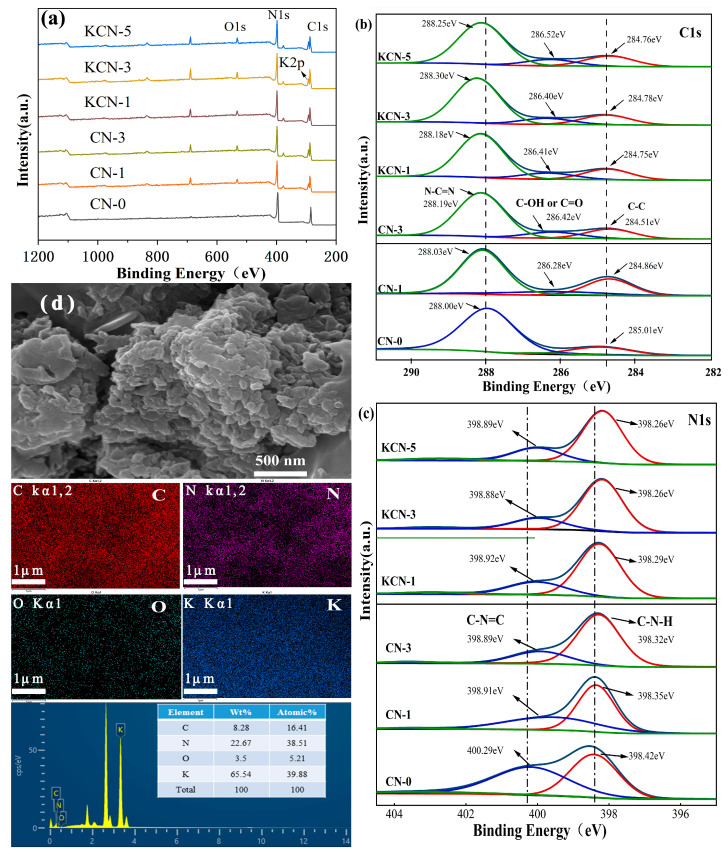
XPS spectra of CN-X and KCN-X: (**a**) full spectrum, (**b**) C1s spectrum, (**c**) N1s spectrum, (**d**) SEM and EDS Mapping of KCN-3.

Figure 3a is the XPS spectra of CN-0, CN-1, CN-3, KCN-1, KCN-3 and KCN-5. It can be seen that these materials all have C, N and O elements. K2p can be seen at about 294 eV for CN-1, CN-3, KCN-1, KCN-3 and KCN-5. Figure 3b is the C1s spectrum of the samples. All materials have two peaks located at 284.8 eV and 288.3 eV, corresponding to C=N and C-NH_2_, respectively. Compared with CN-0, CN-1, and CN-3, KCN-1, KCN-3 and KCN-5 showed a new peak near 286.4 eV, which is speculated to correspond to the binding energy of C or C=O connected to hydroxyl groups [32], which is basically consistent with the detection results of FT-IR. Figure 3c is the N1s spectrum of the sample, which is fitted with two peaks centered at about 398.4 eV and 400.2 eV, corresponding to sp2 hybridized N (C-N-C) and N atoms on free amino groups (C-N-H), respectively. Compared with CN-0, the binding energy of the KCN-X and CN-X peaks are lower, which might be attributed to the fact that -OH acts as an electron-donating group due to the conjugation effect, thereby increasing the electron cloud density around the N atom and reducing the binding energy [33]. From Figure 3d, KCN-3 exhibits a typical amorphous layer structure with a smooth surface and irregularly shaped block stacking structure, which indicates that potassium doping has not changed the original morphology of g-C_3_N_4_. The EDS mapping reflects the uniform distribution of C, N, K, and O elements. This elemental composition agrees well with the XPS survey spectra in Figure 3a. Specifically, the K2p peak appears at ~294 eV, consistent with NIST database values, confirming the successful incorporation of K⁺ into the g-C_3_N_4_ framework.

**Figure 4:**
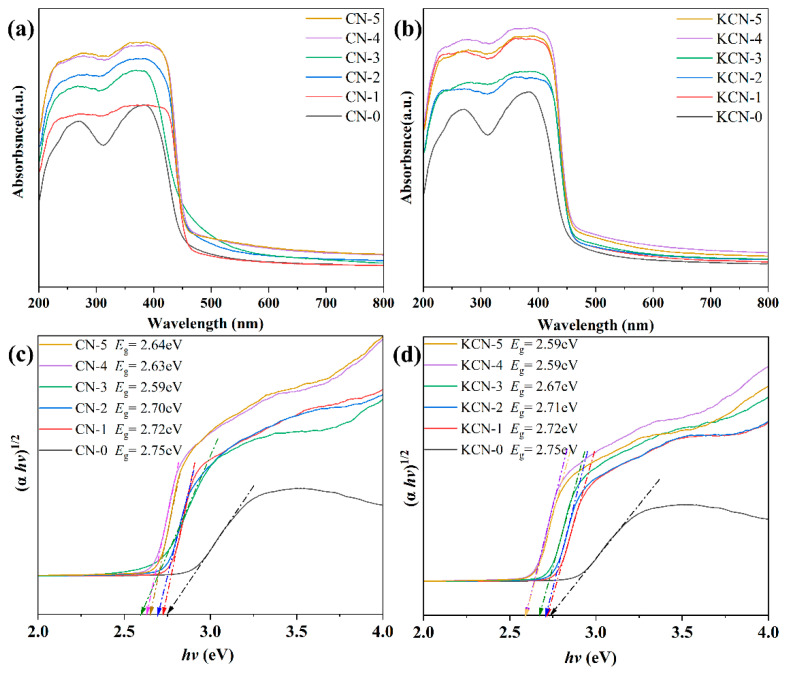
(**a**,**b**) UV–visible diffuse reflectance spectra and (**c**,**d**) optical band gap distribution of CN-X and KCN-X.

As shown in Figure 4a,b, the absorption edges of CN-X (prepared with different KCl and melamine ratios) and KCN-X (treated with different KOH concentrations) are both located at about 465 nm. Compared with the original g-C_3_N_4_ (CN-0 and KCN-0), both absorption edges of CN-X and KCN-X show different degrees of red shift, indicating that their visible light absorption ability is enhanced. Specifically, the absorption edge of the CN-X shows a trend of first red shift and then blue shift with the increase in the KCl ratio, and CN-3 (KCl: melamine = 3:1) shows the largest red shift. The absorption edge of the KCN-X gradually red shifts with the increase in KOH concentration and then stabilizes after KCN-4. Furthermore, the absorption edge was fitted based on the Kubelka-Munk function to obtain the optical band gap (Figure 4c,d). The results show that the band gap of CN-X first decreases and then increases with the increase in the KCl ratio, and the band gap of CN-3 is the smallest. The band gap of KCN-X gradually decreases with the increase in the KOH concentration and tends to be stable after KCN-4. The reduction in band gap not only improves the absorption efficiency of the material for visible light but also enhances its redox ability, which is conducive to the production of active species such as O_2_^−^ and OH under photoexcitation conditions [34], thereby promoting the photocatalytic degradation reaction of the lignin model compound.

### 3.2. Photoelectric Performance Test Experiment

**Figure 5:**
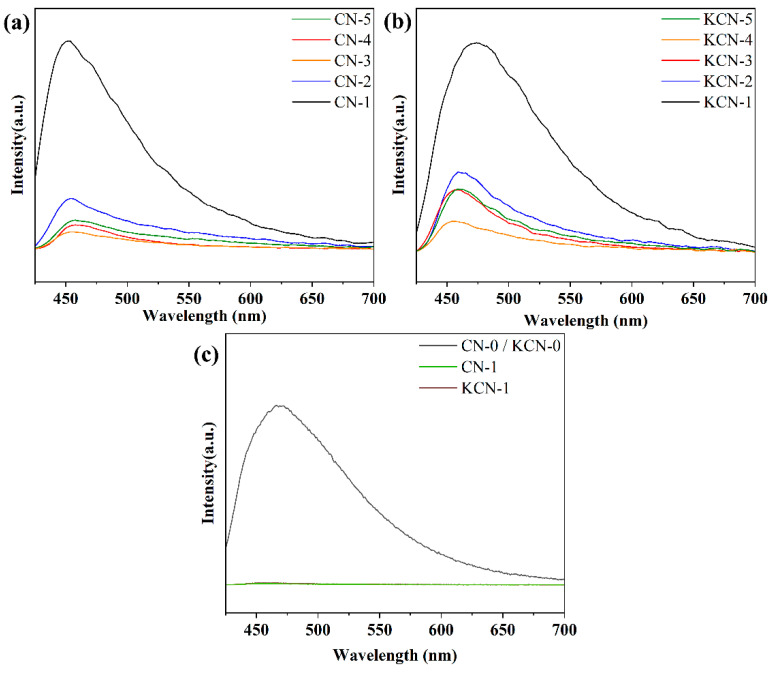
PL spectra of (**a**) CN-X, (**b**) KCN-X, and (**c**) CN-1, KCN-1 and CN-0/KCN-0.

As shown in Figure 5, the photoluminescence (PL) intensity of CN-X and KCN-X is significantly lower than that of unmodified g-C_3_N_4_ (CN-0/KCN-0), and there is almost no obvious luminescence peak at 465 nm, indicating that the recombination of photogenerated electrons and holes is effectively suppressed. This result shows that CN-X and KCN-X after alkalization have higher carrier separation efficiency and lower recombination probability under visible light irradiation, thereby improving their photocatalytic performance. It is found that the PL intensity of CN-X samples first decreased and then increased with the increase in the KCl ratio, among which CN-3 (KCl:melamine = 3:1) showed the lowest PL intensity, indicating that its carrier separation ability is the strongest. Similarly, the PL intensity of the KCN-X gradually decreases with the increase in the KOH concentration, reaching the minimum at KCN-4 (2 mol/L), and then slightly recovers. The results show that the appropriate KCl doping and KOH treating can significantly inhibit the recombination of electron-hole pairs, optimize the photogenerated carrier dynamics of the material, and thus enhance its photocatalytic activity [35].

**Figure 6:**
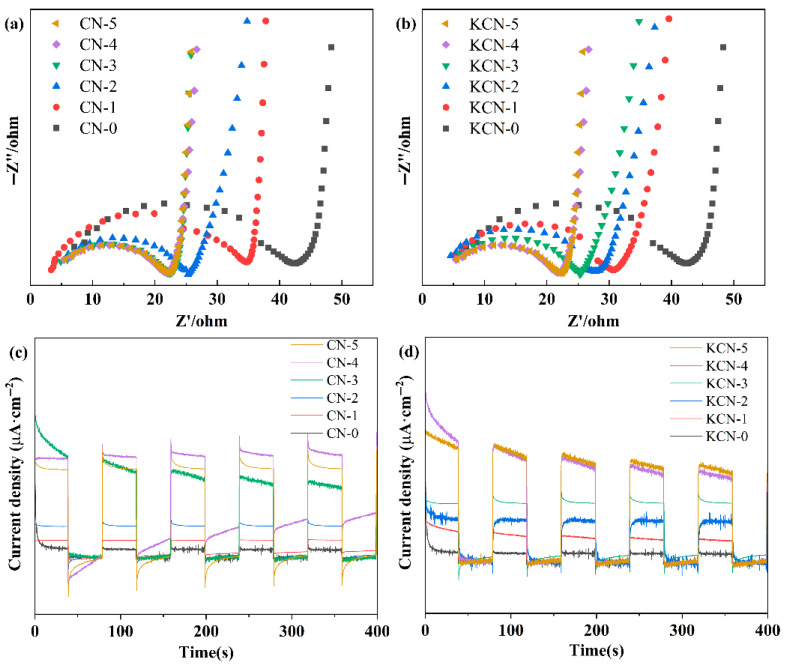
(**a**,**b**) EIS and (**c**,**d**) transient photocurrent of CN-X and KCN-X.

The electrochemical impedance and transient photocurrent of CN-X prepared with different KCl and melamine ratios and KCN-X prepared with different KOH concentrations were tested by an electrochemical workstation under simulated sunlight irradiation. The results are shown in Figure 6. As seen from Figure 6a,b, surface alkalinization induced an obvious decline in the charge transfer resistance (R_ct_) for both CN-X and KCN-X samples, as derived from the diameter of the high-frequency semicircle in Nyquist plots. The charge transfer resistance of CN-X first decreases and then remains unchanged with the increase in KCl proportion, and the KCN-X exhibits a similar change trend with the increase in the KOH concentration. The results indicated that the alkalinization process effectively reduced the interface electron transfer resistance of C_3_N_4_. As shown in Figure 6c,d, the transient photocurrents of CN-X and KCN-X increase after alkalization. Figure 6c shows that the transient photocurrent of CN-X first increases and then decreases with the increase in KCl proportion. Among them, the transient photocurrent of CN-3 is the largest. Figure 6d shows that the transient photocurrent of KCN-X first increases and then remains unchanged with the increase in the KOH concentration. This is consistent with the results of electrochemical impedance.

### 3.3. Catalytic Activity of Alkalinized G-C_3_N_4_ on Lignin Model Compound

**Figure 7:**
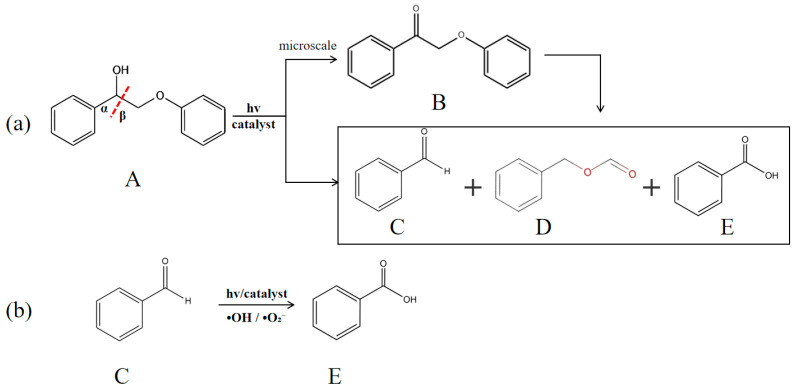
Proposed photocatalytic cleavage and oxidation pathways of 2-phenoxy-1-phenylethanol. (**a**) Under visible-light irradiation, the β-O-4 model compound is depolymerized into the main products benzaldehyde, benzoic acid, and benzyl formate, along with a trace amount of 2-phenoxy-1-phenylethanone. (**b**) Benzaldehyde is further oxidized to benzoic acid through the synergistic effect of photocatalysis and reactive oxygen species (OH, O_2_^−^).

**Figure 8:**
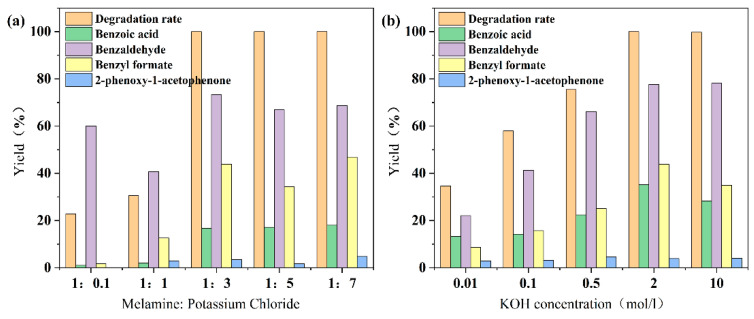
Effect of (**a**) CN-X and (**b**) KCN-X on the degradation of lignin model compound.

In order to evaluate the photocatalytic performance of CN-X and KCN-X, the 2-phenoxy-1-phenylethanol dimer model compound was used as the target, and the degradation was carried out under visible light. As shown in Figure 7a, the possible oxidation products of 2-phenoxy-1-phenylethanol include 2-phenoxy-1-acetophenone (B), benzaldehyde (C), benzyl formate (D) and benzoic acid (E) [36]. All samples taken from the degradation experiment showed that the above four substances were present. The degradation results of 2-phenoxy-1-phenylethanol by CN-X and KCN-X are shown in Figure 8.

As shown in Figure 8, after alkalization, the depolymerization effect of CN-X and KCN-X on 2-phenoxy-1-phenylethanol has been significantly improved. Figure 8a shows that with the increase in KCl proportion, the degradation rate of 2-phenoxy-1-phenylethanol gradually increases. When the ratio of melamine to KCl changes to 1:3, the degradation rate increases to 100%, which is consistent with the EIS and PL test results. The increase in K^+^ reduces the band gap of the material (Figure 4) and the interface electron transfer resistance, improves the redox ability of the material, and promotes the generation of O_2_^−^ and OH. However, as the ratio of melamine to KCl further changes to 1:5 and 1:7, the degradation rate remains stable.

Figure 8b shows that the degradation rate of 2-phenoxy-1-phenylethanol gradually increases with the increase in KOH concentration. When the KOH concentration increases to 2 mol/L, the degradation rate increases to 100%. Combined with the results of XRD and FTIR, the increase in KOH concentration allows the material surface to have more abundant hydroxyl groups, thereby optimizing the band structure of g-C_3_N_4_ (Figure 4) and making the material have stronger redox ability. However, when the KOH concentration increases to 10 mol/L, the degradation rate remains at 100%, indicating that too much KOH no longer has a beneficial effect on the degradation of 2-phenoxy-1-phenylethanol.

**Figure 9:**
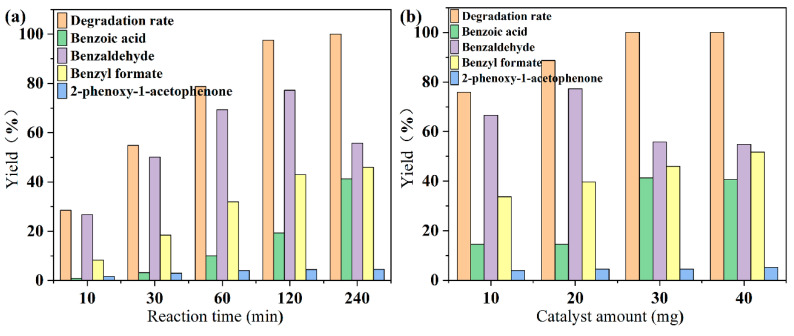
Effects of different (**a**) reaction time and (**b**) catalyst amount on the degradation of lignin model compound.

Combining the depolymerization effects of the two kinds of materials on 2-phenoxy-1-phenylethanol and the previous characterization analysis, KCN-4 was chosen for the subsequent study. The effects of reaction time and catalyst amount on the degradation performance, including the 2-phenoxy-1-phenylethanol degradation rate and its product yield, are shown in Figure 9.

As seen in Figure 9a, before 120 min, the degradation rate of 2-phenoxy-1-phenylethanol and its product yields gradually increased. At 120 min, the degradation rate of 2-phenoxy-1-phenylethanol reached 97.2%, indicating that there was still some substrate left. And the yield of benzaldehyde reached a maximum of 77.28%. At 240 min, the degradation rate of 2-phenoxy-1-phenylethanol reached 100%, but the yield of benzaldehyde decreased to 55.79%. On the contrary, the yields of benzyl formate and benzoic acid increased to 45.99% and 41.32%, respectively. It can be inferred that the increase in the yield of benzyl formate may be due to the continued degradation of the remaining 2-phenoxy-1-phenylethanol or the degradation of 2-phenoxy-1-acetophenone (B). To confirm the degradation pathway, we conducted a set of degradation experiments with 2-phenoxy-1-acetophenone as the substrate. The results are shown in Table 2. The KCN-4 exhibited a limited degradation effect on 2-phenoxy-1-acetophenone (only 5.87%), and the amount of 2-phenoxy-1-acetophenone obtained by the depolymerization of 2-phenoxy-1-phenylethanol is very small. After 120 min of reaction, the amount of 2-phenoxy-1-acetophenone is almost unchanged. Therefore, the increase in the yield of benzyl formate is obtained by the continued degradation of the remaining 2-phenoxy-1-phenylethanol. The increase in the benzoic acid yield can be inferred to be caused by the photocatalytic oxidation of benzaldehyde to produce benzoic acid after the complete depolymerization of 2-phenoxy-1-phenylethanol. To confirm this, a set of degradation experiments with benzaldehyde as the substrate was conducted, and the results are shown in Table 2. Benzaldehyde can be completely oxidized to benzoic acid. In summary, the selectivity of KCN-4 for C_α_-C_β_ bonds is prior to -CHO during photocatalytic reaction, so the yield of benzoic acid can be controlled by regulating the reaction time.

In Figure 9b, the degradation rate of 2-phenoxy-1-phenylethanol gradually increases with the increase in catalyst amount. When the catalyst is increased to 30 mg, the yield reaches 100% after 240 min of reaction. However, the yield of benzaldehyde decreases after the catalyst is increased to 30 mg. Combined with the previous analysis, it can be inferred that when the amount of catalyst is 10 mg and 20 mg, the substrate cannot be completely depolymerized at 240 min of reaction. Therefore, the amount of benzaldehyde is increasing all the time. When the catalyst was increased to 30 mg, the substrate was completely depolymerized within 240 min of reaction, and the previously generated benzaldehyde was further oxidized to benzoic acid. The selectivity toward benzaldehyde originates from the preferential cleavage of the C_β_–O bond in β-O-4 linkages, which has a lower bond dissociation energy compared to C_α_-C_β_. Under visible-light irradiation, photogenerated holes on g-C_3_N_4_ facilitate this bond cleavage. As the reaction proceeds, benzaldehyde can be further oxidized to benzoic acid through the action of reactive oxygen species (ROS) such as OH or O_2_^−^, especially in the presence of surface hydroxyl groups introduced by KOH treatment. This stepwise mechanism explains the observed selectivity shift from benzaldehyde to benzoic acid with increasing reaction time or catalyst dosage. The alkalinized surface modulates the oxidation potential, enabling controllable oxidative cleavage and product distribution. These mechanistic insights are schematically illustrated in Figure 7, which outlines the selective bond cleavage, oxidation pathways, and the formation of major and minor products. Therefore, for the depolymerization of 2-phenoxy-1-phenylethanol by KCN-4, we can control the yield of benzaldehyde and benzoic acid by adjusting the catalyst amount and the reaction time.

**Figure 10:**
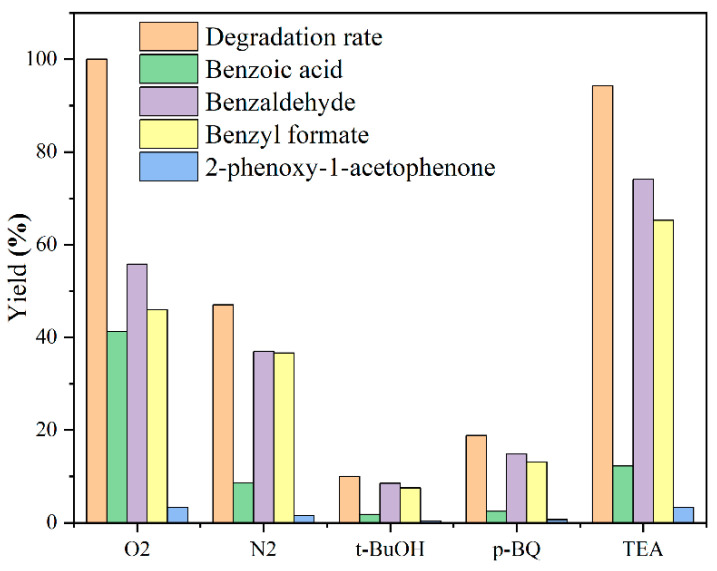
Effect of reaction atmosphere and free radical quencher on the degradation of lignin model compound.

In order to explore the effects of reaction atmosphere and generated free radicals on the photocatalytic process, the comparative experiments were conducted on the conversion of the lignin model compound (Figure 10). The results showed that under an N_2_ atmosphere, the degradation rate and product yield decreased significantly, indicating that O_2_ participated in the reaction process. The reaction almost stopped after the addition of the OH quencher t-BuOH, confirming that OH also plays an important role in the system. After the introduction of the O_2_^−^ quencher p-BQ, the yield was greatly reduced, indicating that O_2_^−^ was also the dominant active free radical. The photogenerated hole quencher TEA had little effect on the reaction, indicating that h⁺ was not the main active species [37,38].

After alkalinization treatment, a large number of nitrogen defects and carbon defects are generated on the surface of the material. These surface defects help to enhance the adsorption capacity of O_2_. Under visible light irradiation, photogenerated holes can react with adsorbed H_2_O and O_2_ to generate hydroxyl radicals (OH) and superoxide radicals (O_2_^−^), which play a key role in the photocatalytic oxidation process. Hence, the g-C_3_N_4_ treated by surface alkalization exhibits excellent photocatalytic performance, which is mainly attributed to two factors. First, Kg-C_3_N_4_ has a high light absorption and utilization ability, enabling it to capture light energy more efficiently. Second, it possesses good photogenerated carrier separation efficiency, which can effectively separate photogenerated carriers and transfer them to the reaction interface, thereby generating OH and O_2_^−^ to promote the degradation reactions.

## 4. Conclusions

This study successfully constructed an efficient g-C_3_N_4_ photocatalyst through a K⁺-doping and surface alkalinization strategy and achieved mild conditions for the depolymerization of the lignin β-O-4 model compound (2-phenoxy-1-phenylethanol). The introduction of KCl destroyed the layered stacking of g-C_3_N_4_. Combined with KOH post-treatment, hydroxyl functional groups were introduced, the band gap was reduced to 2.35 eV and the carrier separation efficiency was improved. The optimized CN-3 and KCN-4 achieved complete substrate degradation under visible light in 240 min, and the benzaldehyde selectivity reached 77.28%. It is found that the surface alkaline sites enhance substrate adsorption through hydrogen bonds, promote the generation of O_2_^−^ and OH, and preferentially break the Cβ-O bond. Regulating the reaction time or catalyst amount can effectively control the product distribution. This work provides a new idea of green catalysis for lignin resource utilization. Its noble-metal-free, recyclable, and tunable catalytic platform shows promise for scalable applications in biomass conversion, especially in producing fine chemicals such as flavors and pharmaceutical intermediates from lignin.

## Figures and Tables

**Table 1:** Sample designation system.

Series	KCl: Melamine (Mass Ratio)	KOH (mol/L)	Sample Label
Baseline	–	–	CN-0/KCN-0
KCl Ratio	0.1:1 *	10	CN-1
	1:1	10	CN-2
	3:1	10	CN-3
	5:1	10	CN-4
	7:1	10	CN-5
KOH Concentration	3:1	0.01	KCN-1
	3:1	0.1	KCN-2
	3:1	0.5	KCN-3
	3:1	2	KCN-4
	3:1	10	KCN-5

* Fixed precursor: 3.0 g melamine.

**Table 2:** The degradation rate and product yield of the depolymerization experiment for 2-phenoxy-1-acetophenone and benzaldehyde.

Substrate	Degradation Rate (%)	Yield (%)		
		Benzoic Acid	Benzaldehyde	Benzoyl Formate
2-phenoxy-1-acetophenone	5.87	28.88	0.12	10.25
Benzaldehyde	100	100	/	/

## Data Availability

The original contributions presented in this study are included in the article. Further inquiries can be directed to the corresponding authors.
